# Supplementary material for: A deep learning framework for understanding cochlear implants
Source: bioRxiv. 2025 Oct 17:2025.07.16.665227. Originally published 2025 Jul 21. Preprint. [Version 2] doi: 10.1101/2025.07.16.665227 (PMC12330565; doi:10.1101/2025.07.16.665227)
Supplement: 1 [file NIHPP2025.07.16.665227v2-supplement-1.pdf]

Supplementary Figure 1. Comparison of word recognition by normal hearing model and normal hearing humans, for speech in noise and noise-vocoded speech.

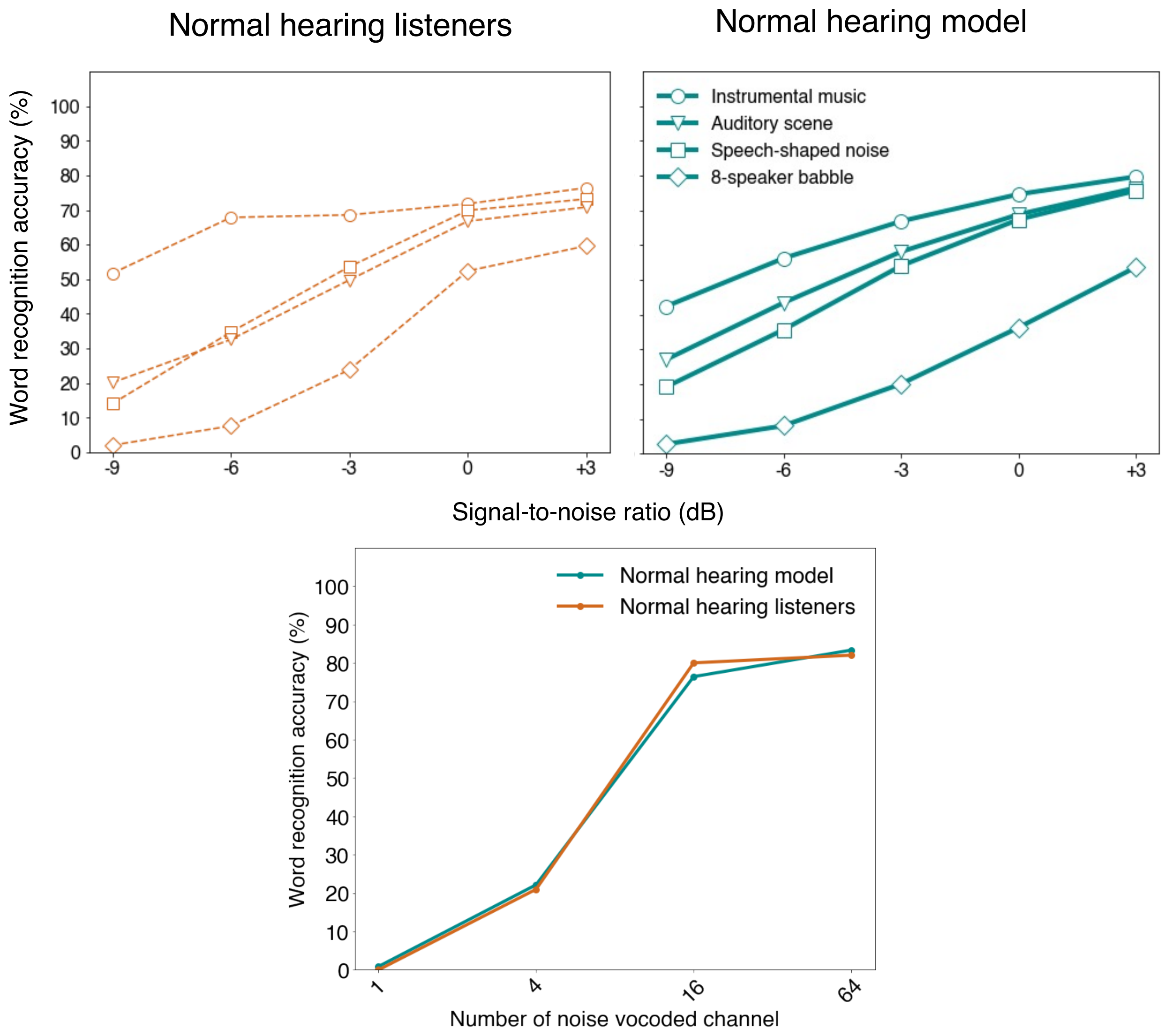

Supplementary Figure 2. Schematic of different sound coding strategies

Advanced Combination Encoder:

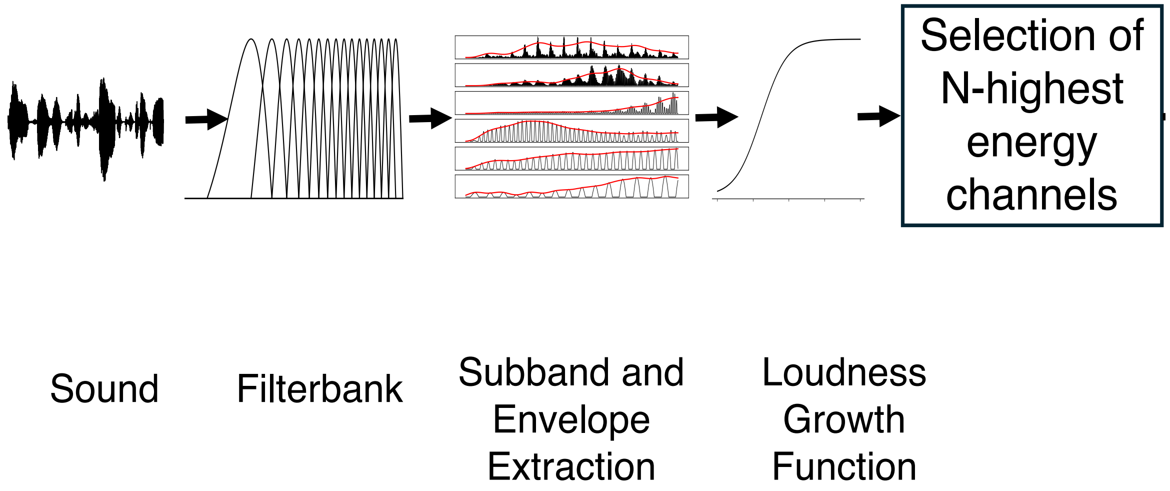

Fine Structure Processing:

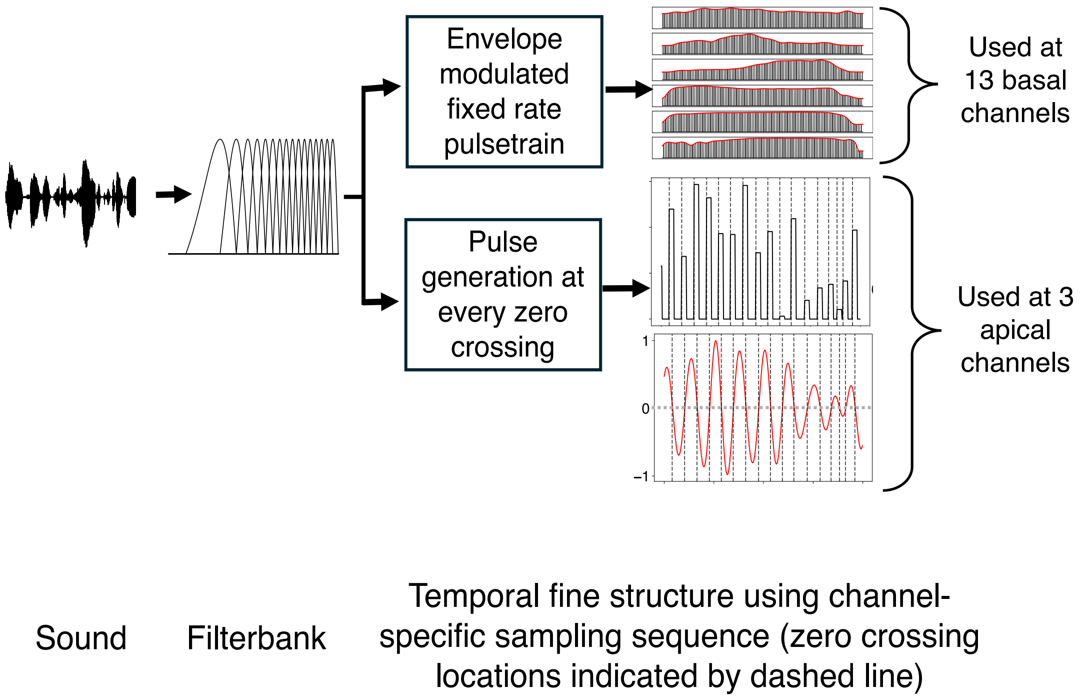

High Resolution with Fidelity 120:

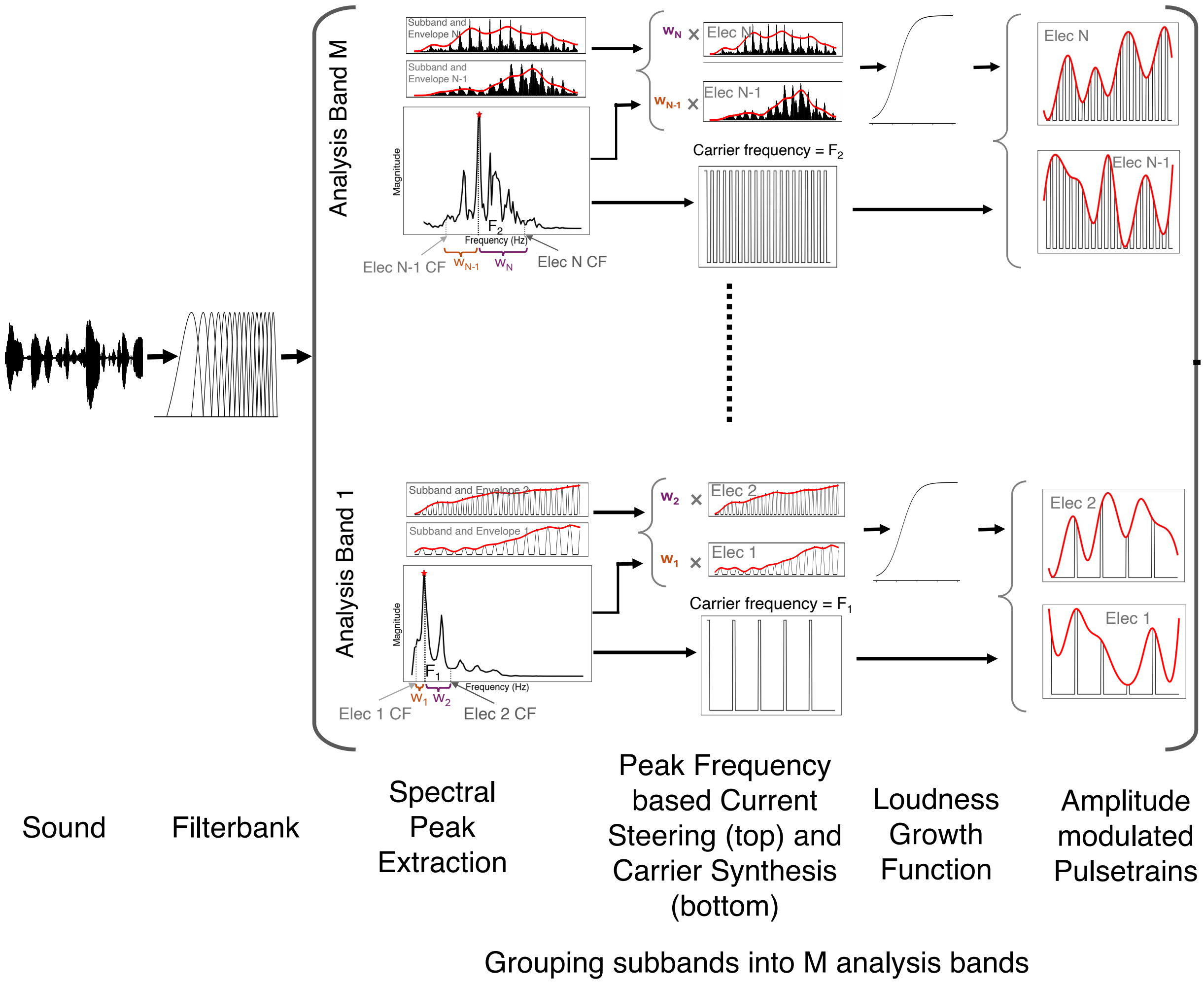

Supplementary Figure 3. Example electrodograms from different sound coding strategies

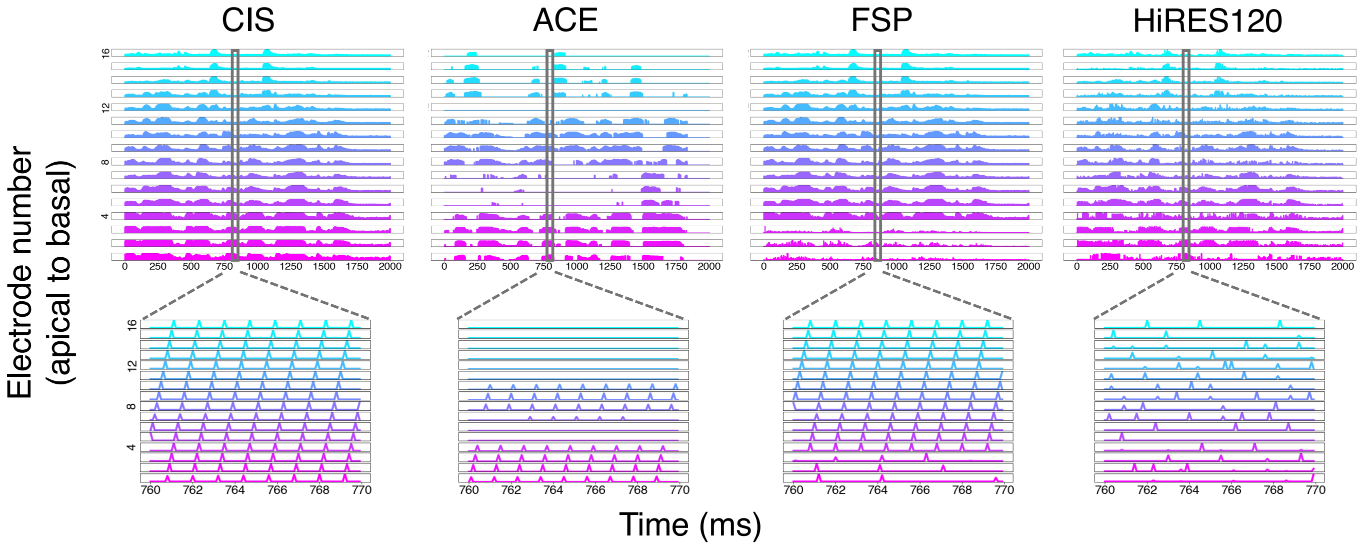

Supplementary Figure 4. ITD-ILD bias for CI models with different sound coding strategies

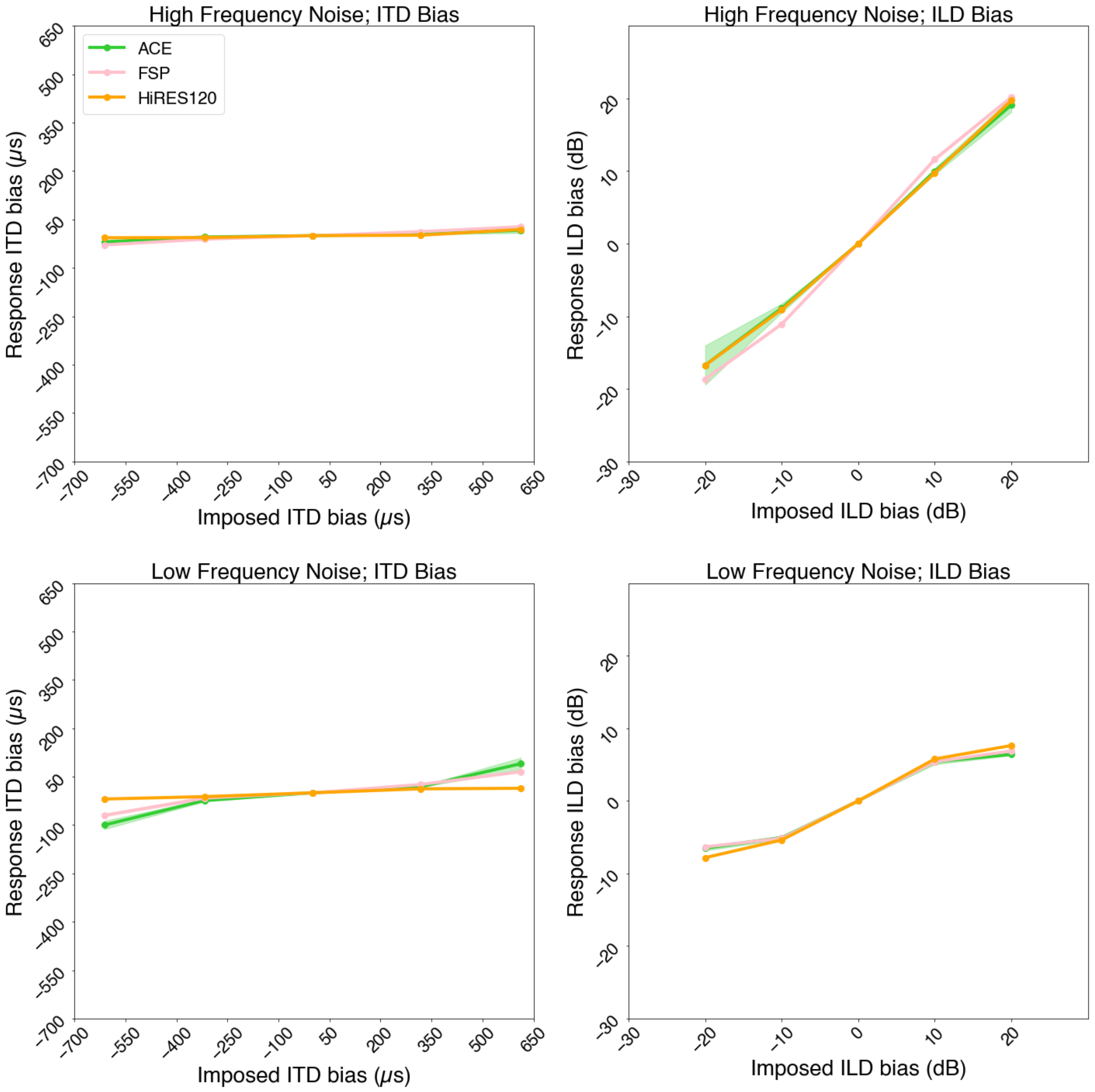

Supplementary Figure 5. Analysis of fine structure ITDs obtained from electrode signals
A. Results for Continuous Interleaved Sampling (CIS)

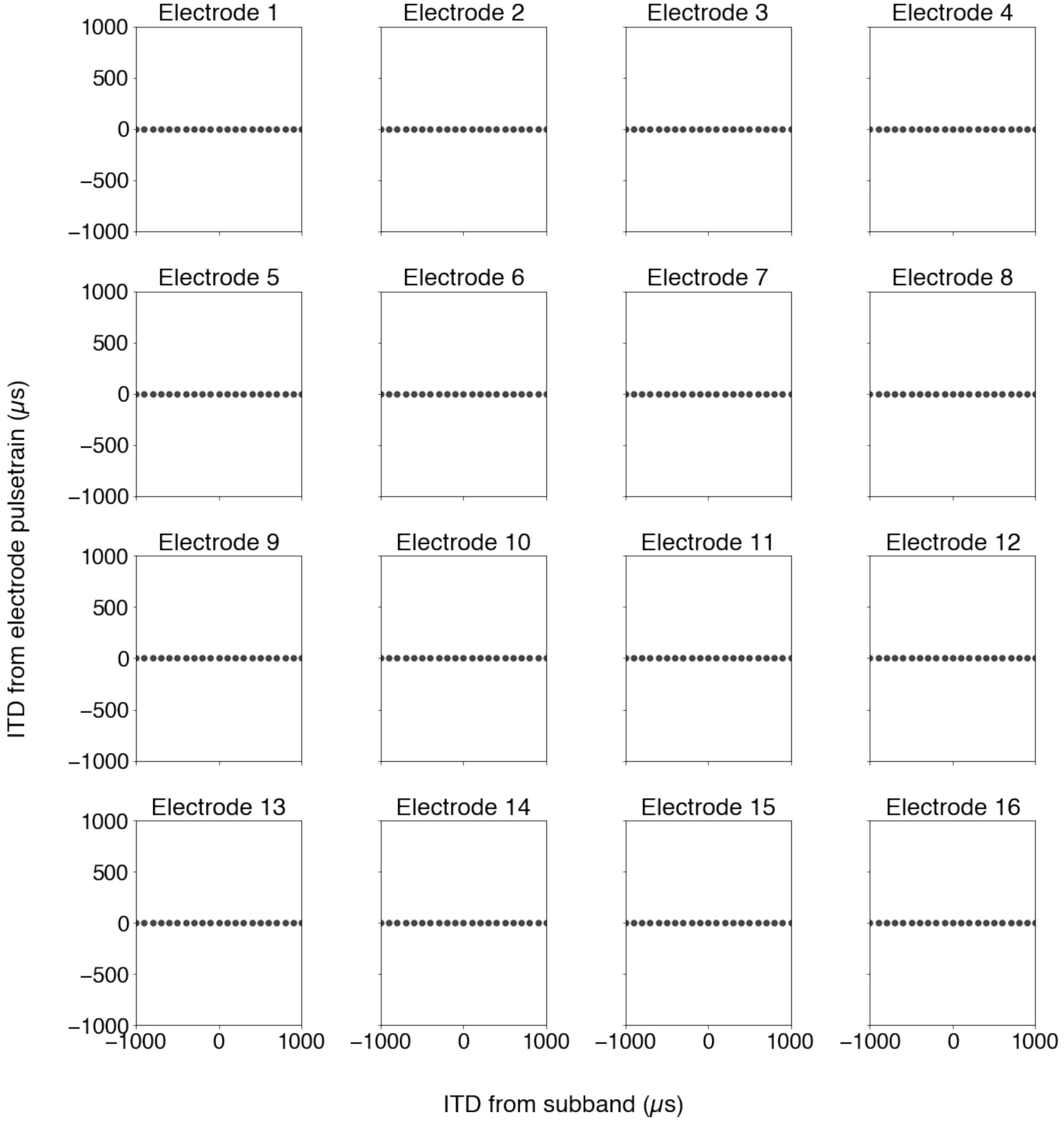

B. Results for Advanced Combination Encoder (ACE)

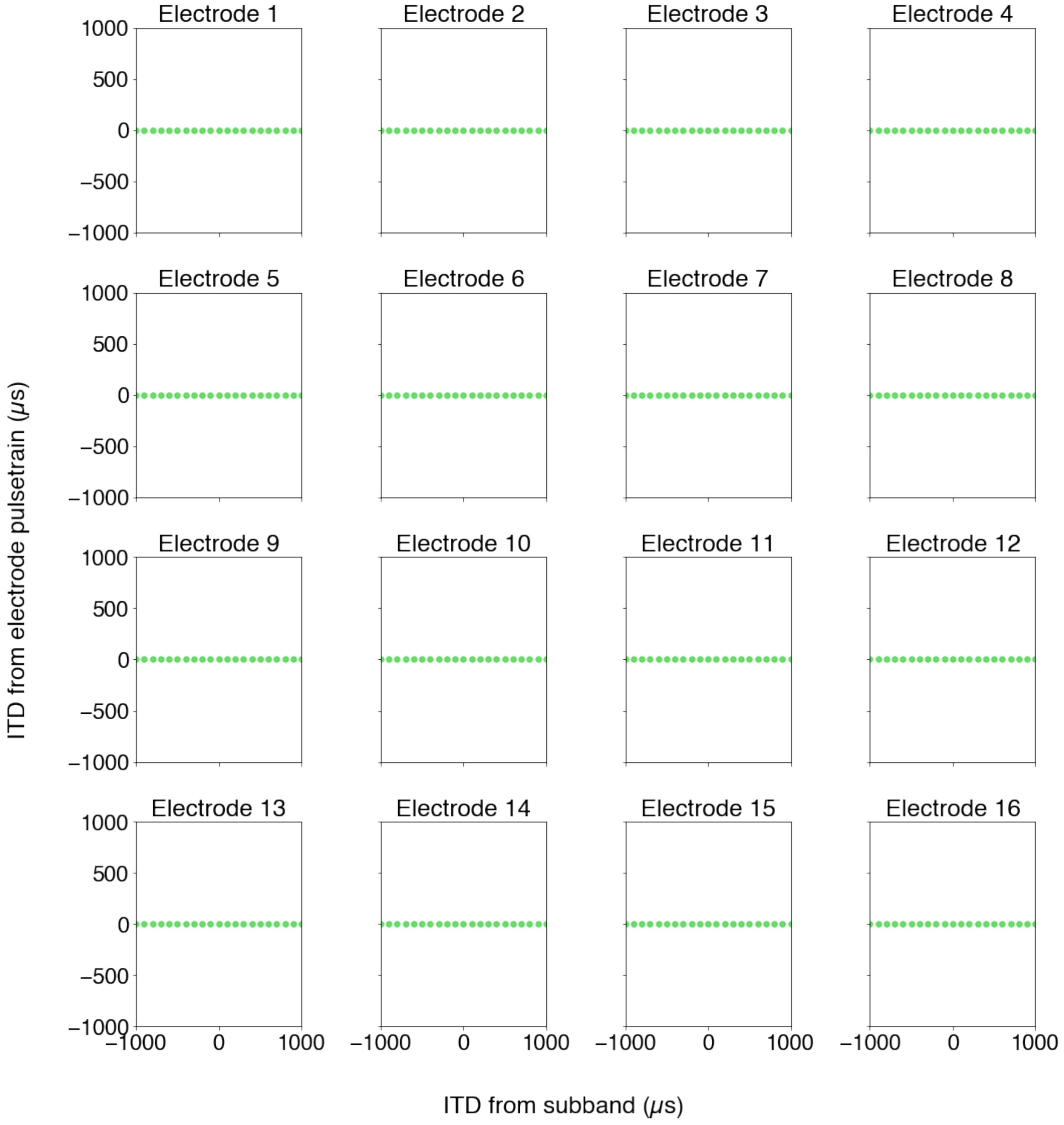

C. Results for Fine Structure Processing (FSP)

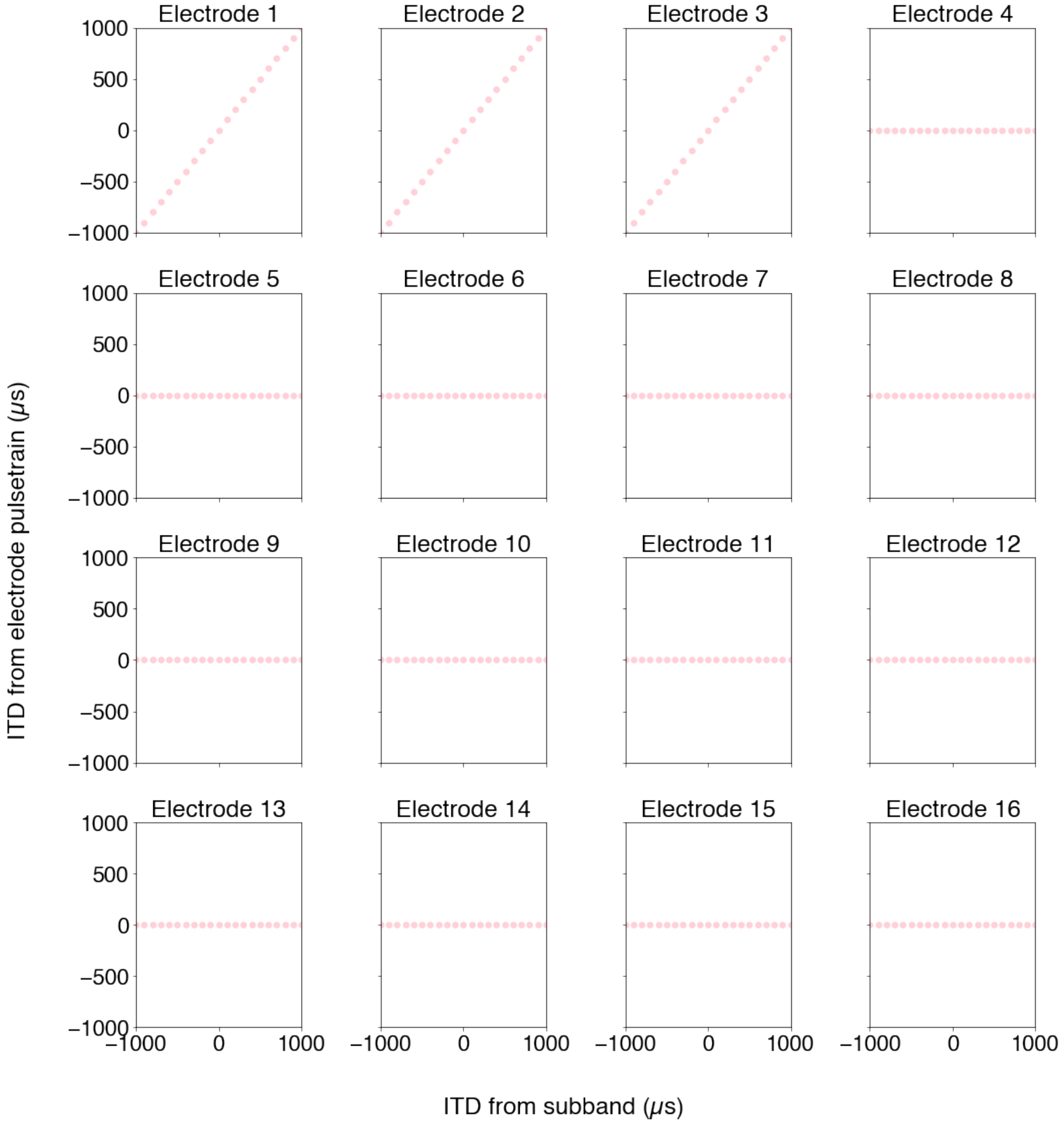

D. Results for High Resolution with Fidelity 120 (HiRES-120)

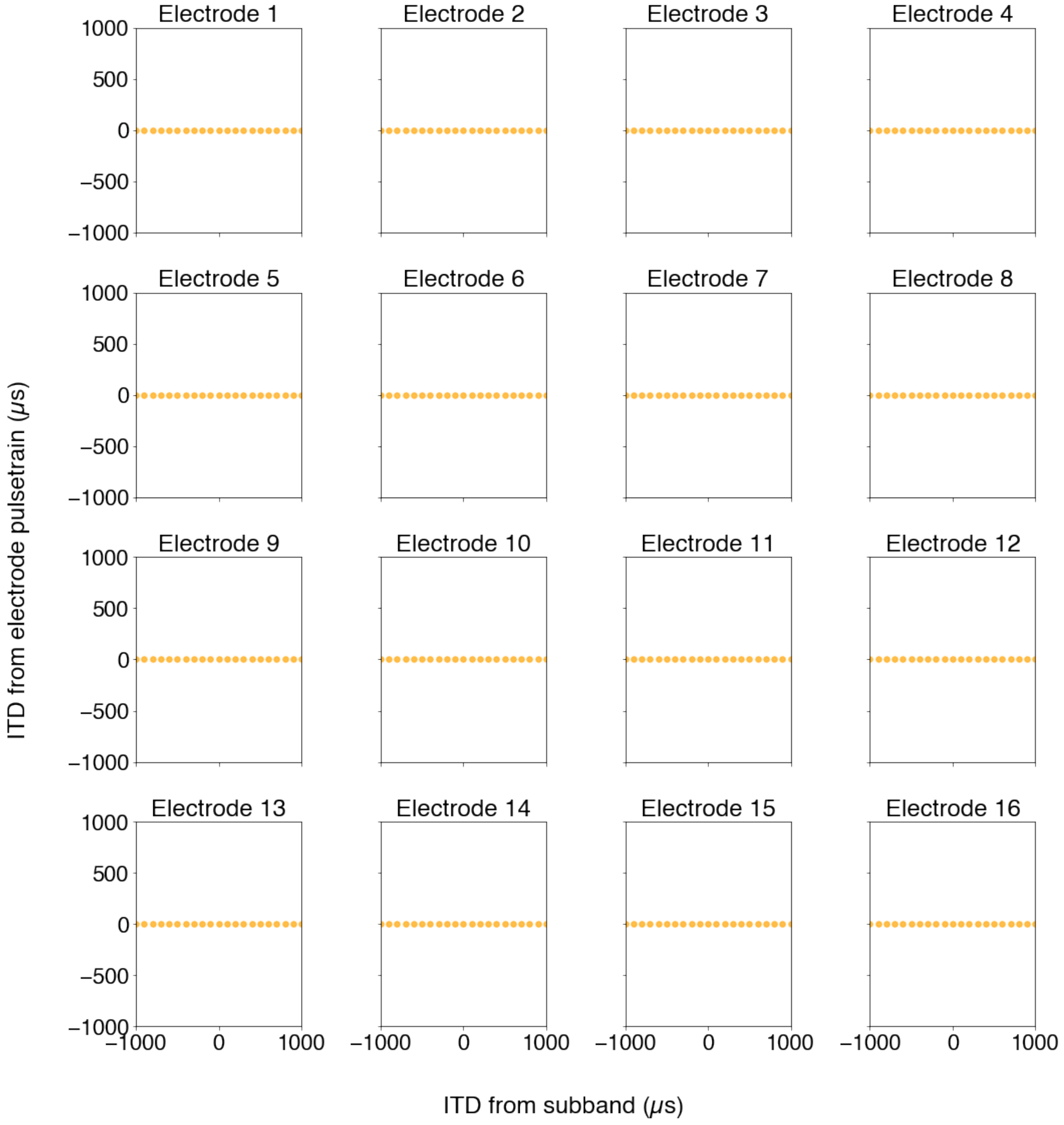

Supplementary Figure 6. Analysis of envelope ITDs obtained from electrode signals
A. Results for Continuous Interleaved Sampling (CIS)

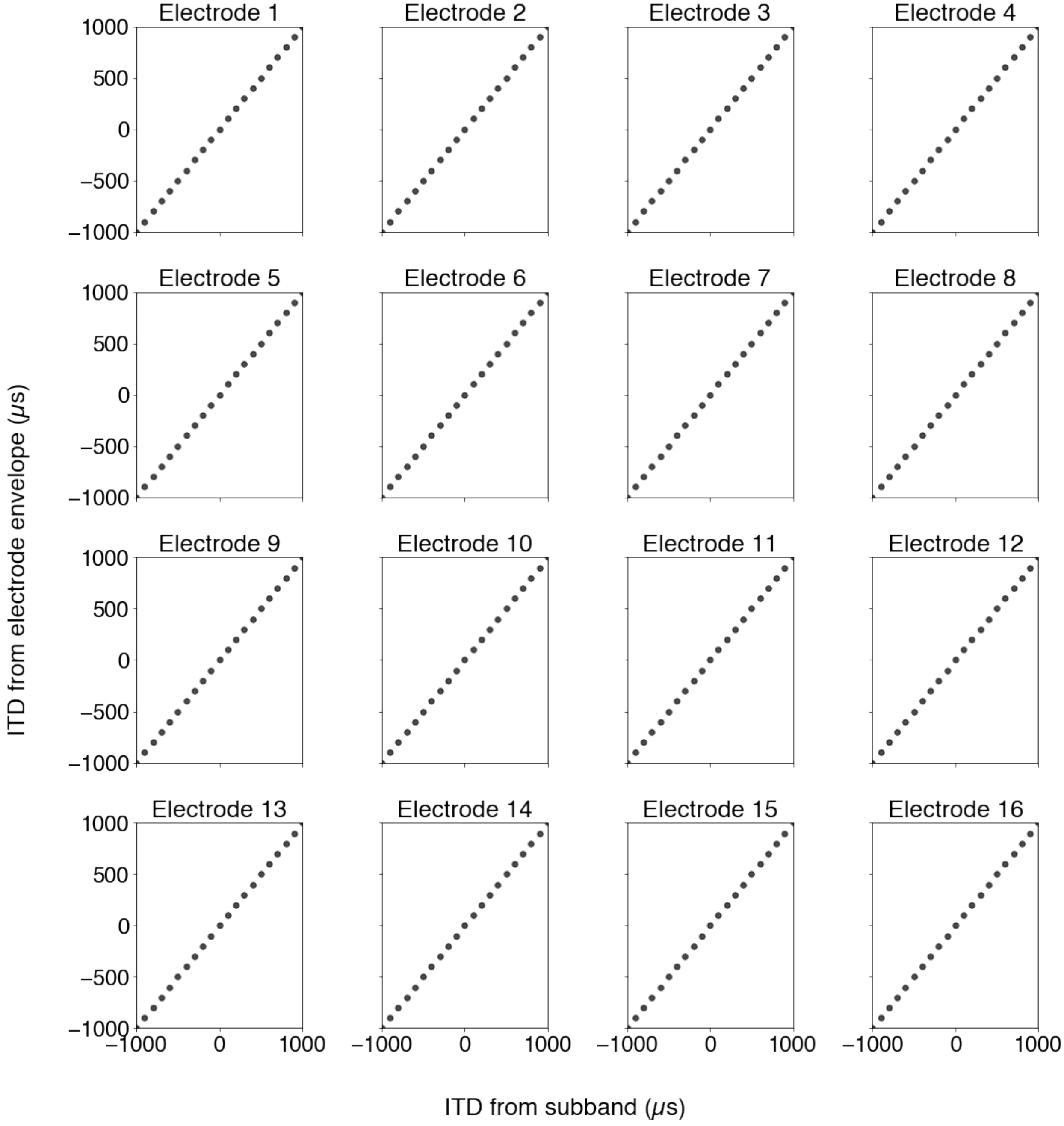

B. Results for Advanced Combination Encoder (ACE)

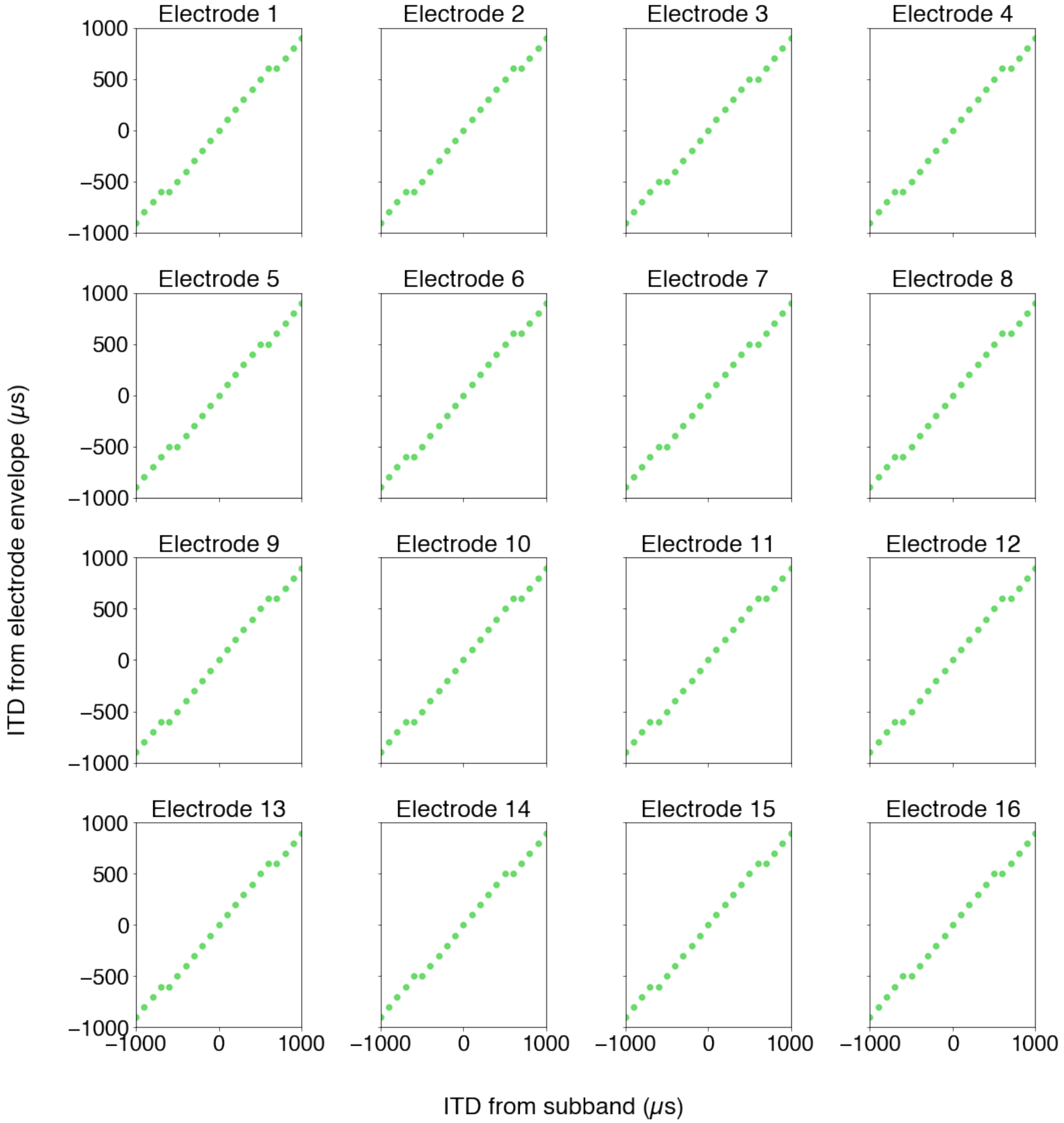

C. Results for Fine Structure Processing (FSP)

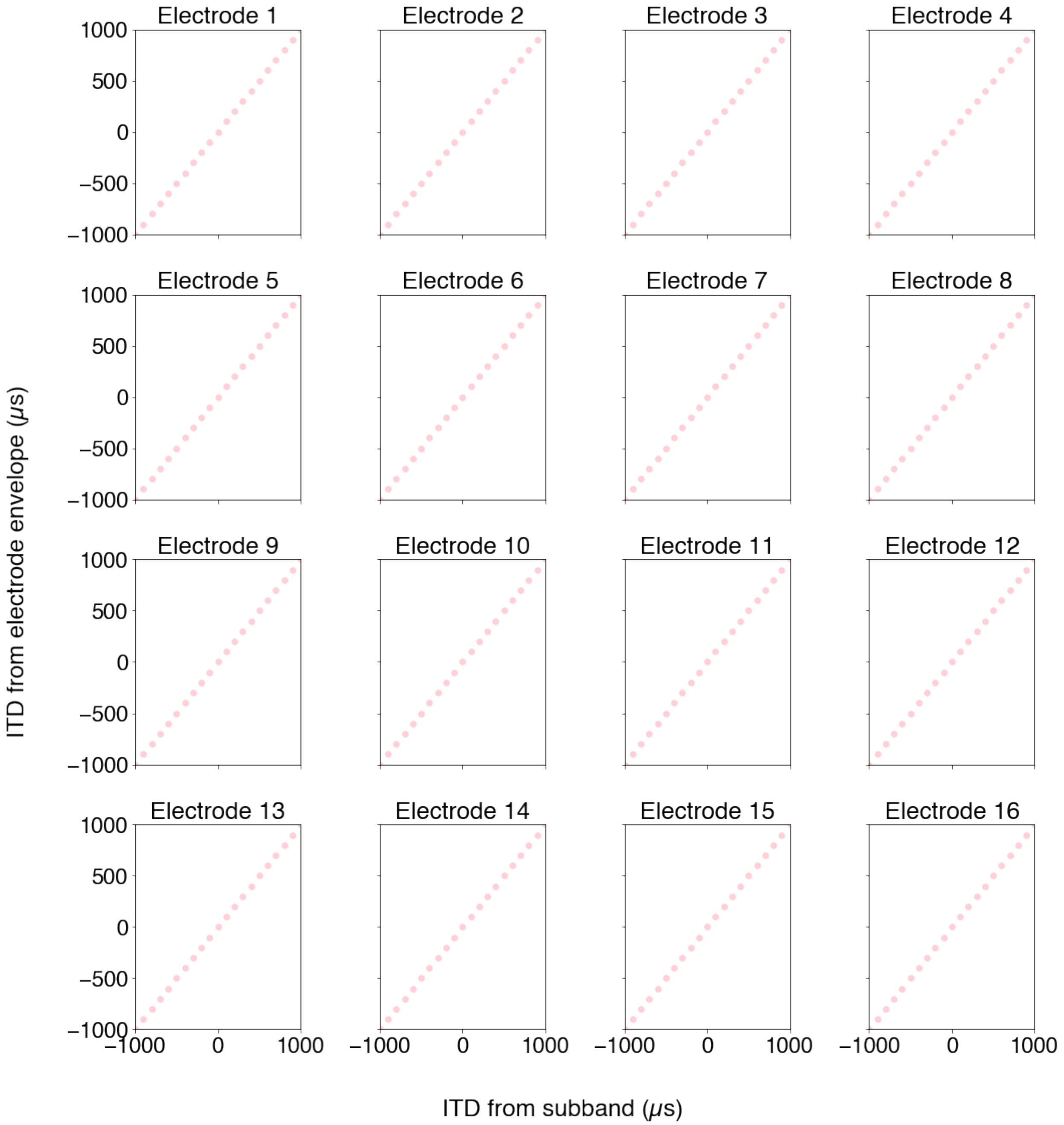

D. Results for High Resolution with Fidelity 120 (HiRES-120)

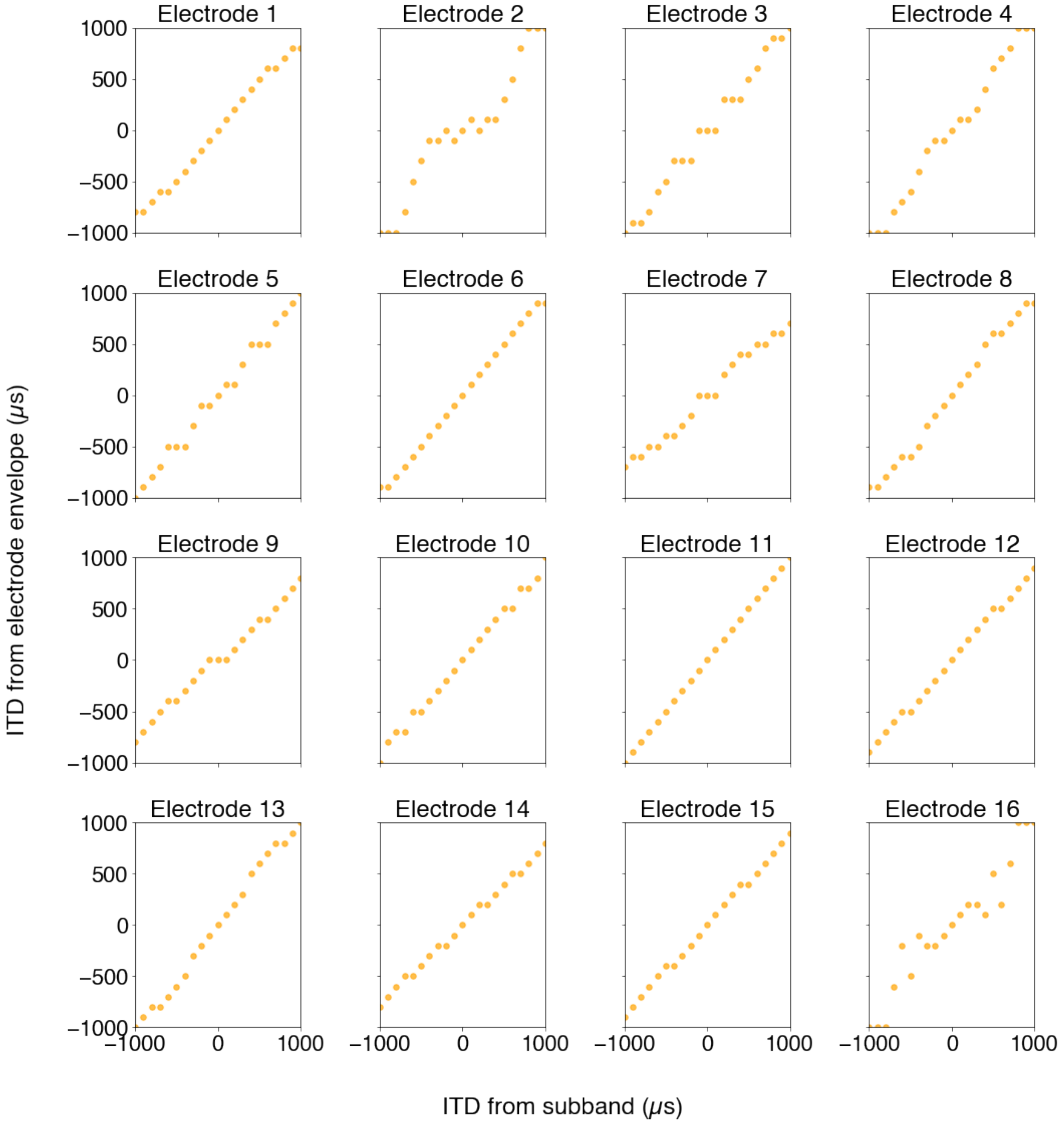

Supplementary Figure 7. Analysis of ILDs obtained from electrode signals
A. Results for Continuous Interleaved Sampling (CIS)

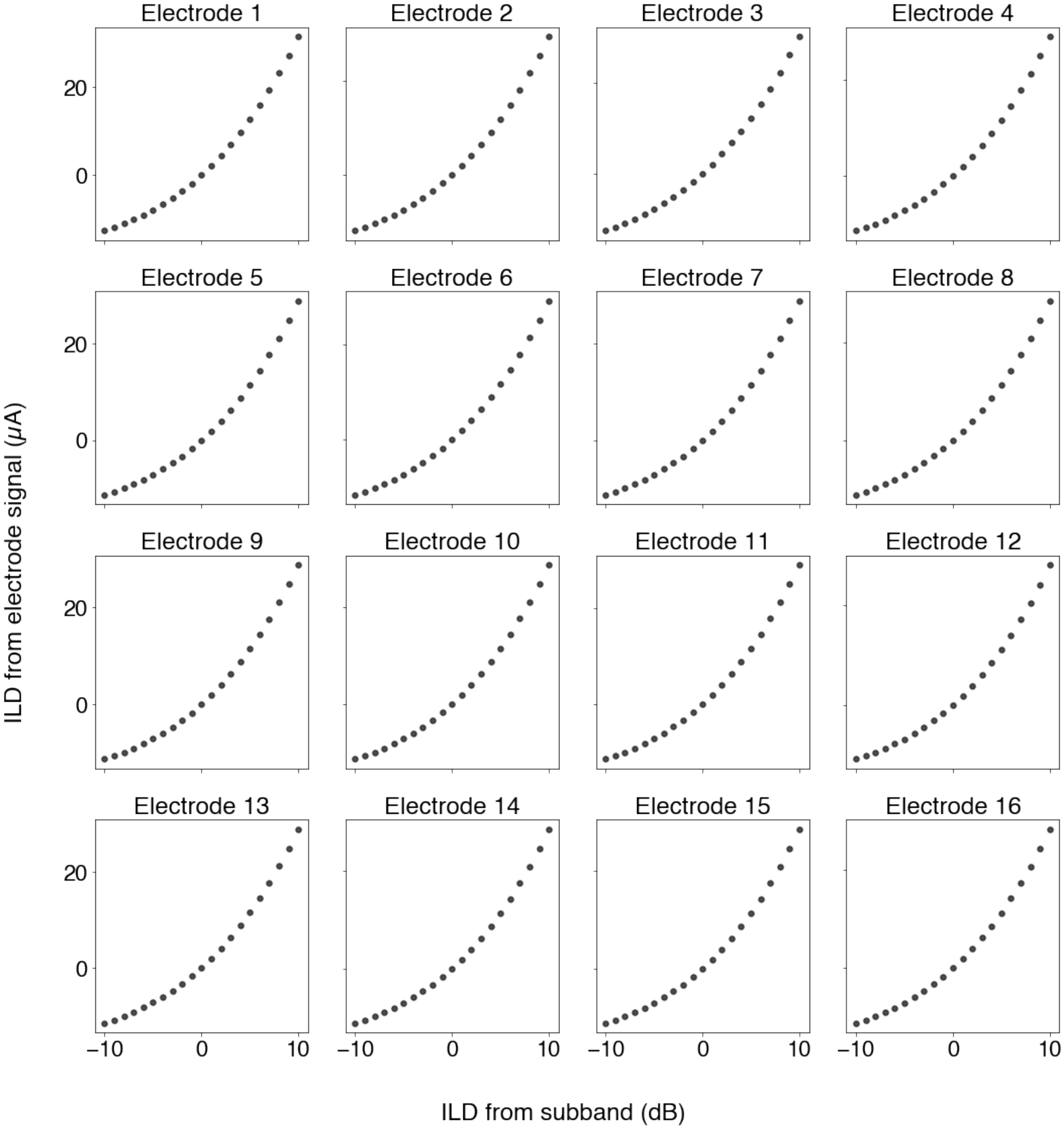

B. Results for Advanced Combination Encoder (ACE)

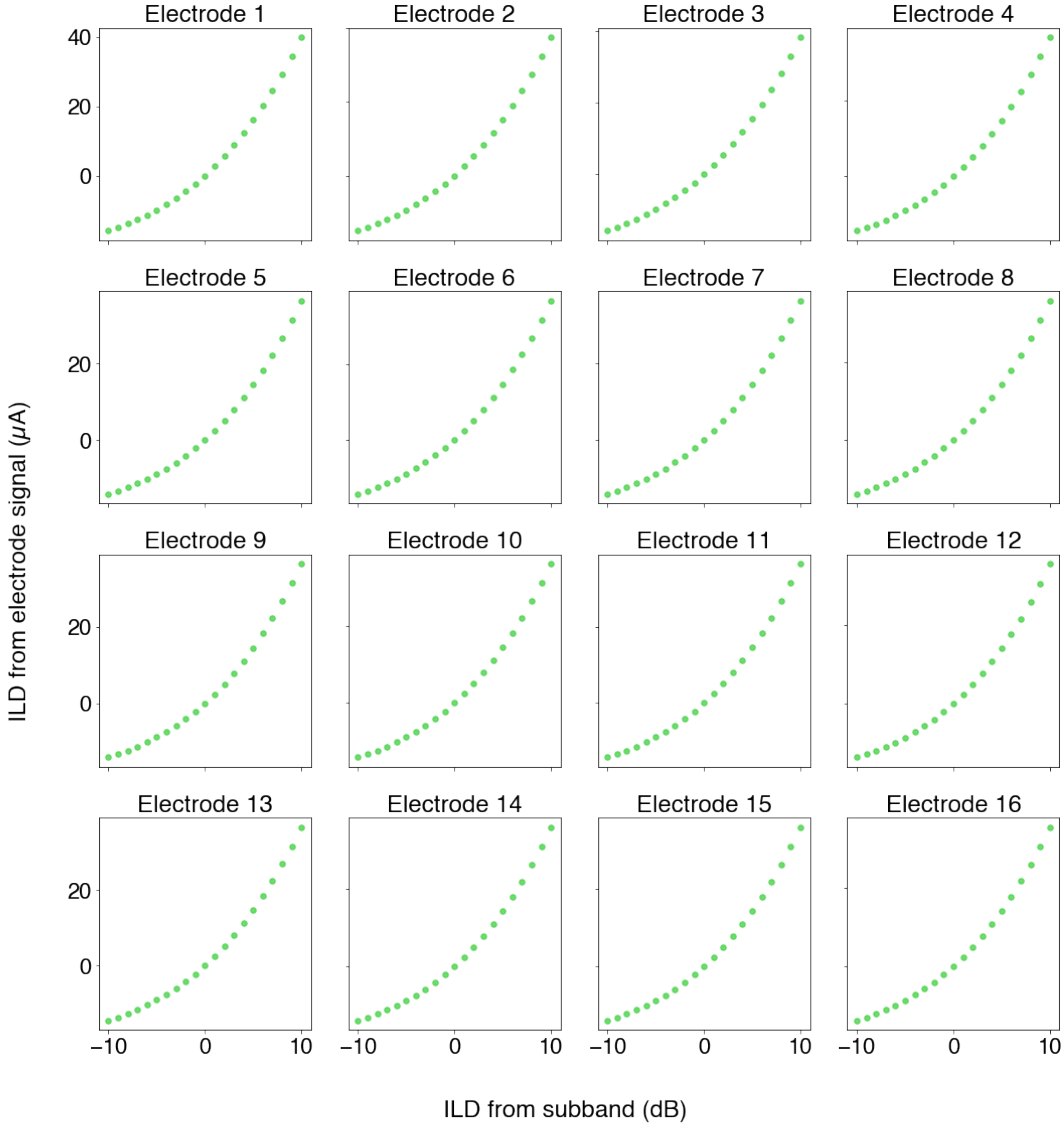

C. Results for Fine Structure Processing (FSP)

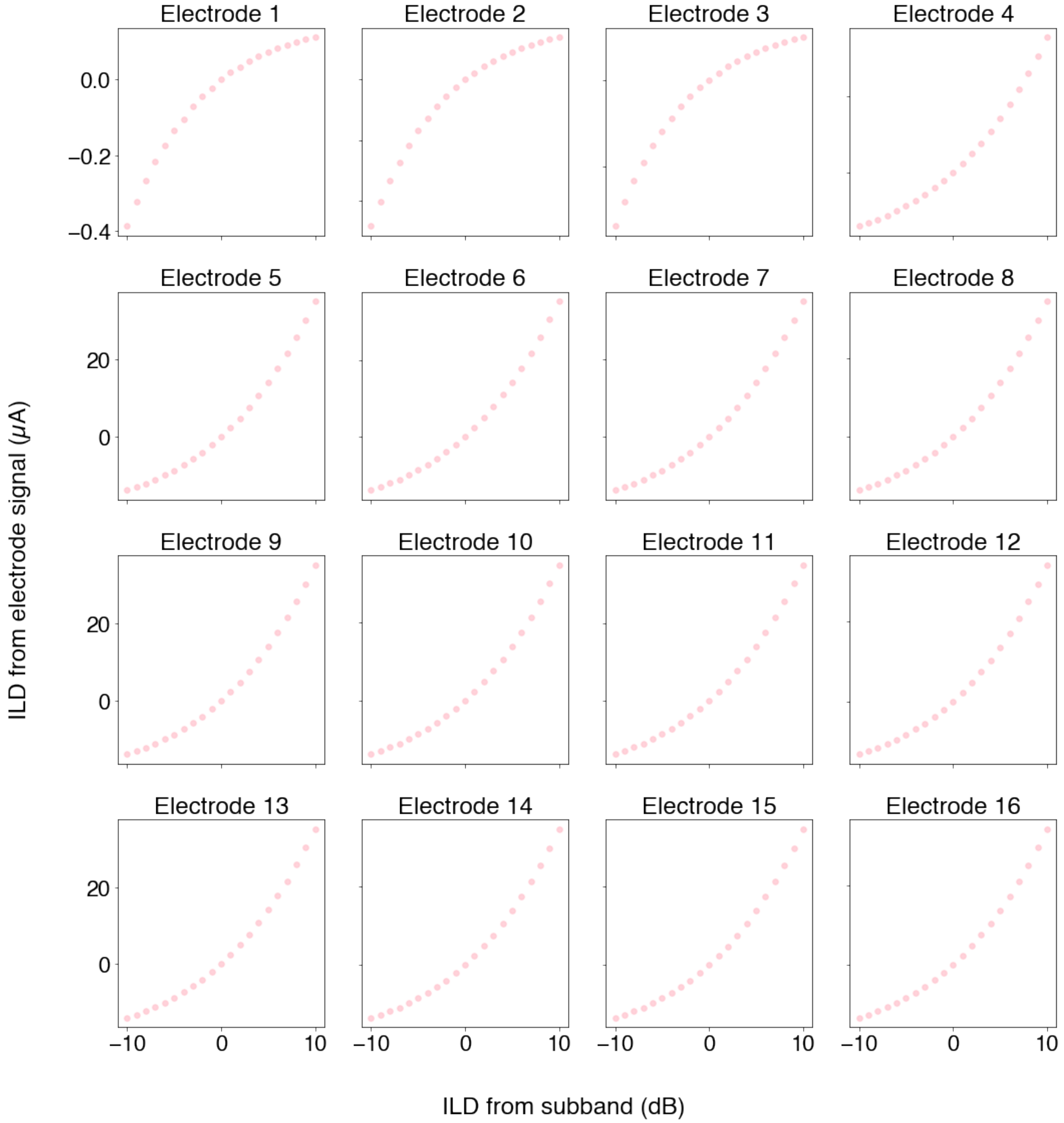

D. Results for High Resolution with Fidelity 120 (HiRES-120)

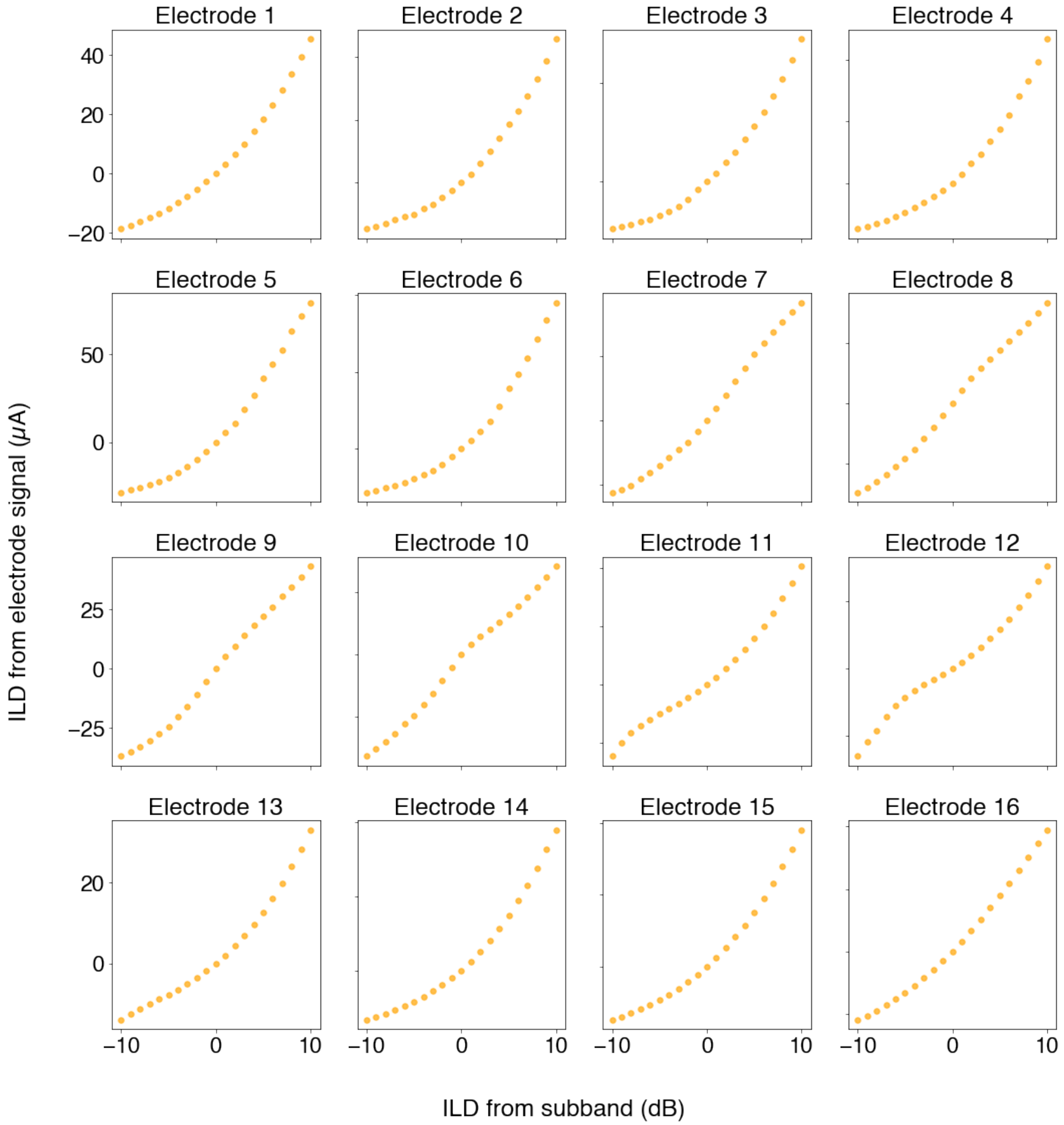

Supplementary Table 1. Details of the three neural network architectures used for the word recognition task

**arch0_0000**	**arch0_0001**	**arch0_0002**
input_lnorm	input_lnorm	input_lnorm
conv0 [2, 42, 32]	conv0 [1, 84, 32]	conv0 [4, 21, 32]
relu0	relu0	relu0
hpool0 [2, 4]	hpool0 [2, 4]	hpool0 [2, 4]
lnorm0	lnorm0	lnorm0
conv1 [2, 18, 64]	conv1 [2, 18, 64]	conv1 [2, 18, 64]
relu1	relu1	relu1
hpool1 [2, 4]	hpool1 [2, 4]	hpool1 [2, 4]
lnorm1	lnorm1	lnorm1
conv2 [6, 6, 128]	conv2 [6, 6, 128]	conv2 [6, 6, 128]
relu2	relu2	relu2
hpool2 [1, 4]	hpool2 [1, 4]	hpool2 [1, 4]
lnorm2	lnorm2	lnorm2
conv3 [6, 6, 256]	conv3 [6, 6, 256]	conv3 [6, 6, 256]
relu3	relu3	relu3
hpool3 [1, 4]	hpool3 [1, 4]	hpool3 [1, 4]
lnorm3	lnorm3	lnorm3
conv4 [8, 8, 512]	conv4 [8, 8, 512]	conv4 [8, 8, 512]
relu4	relu4	relu4
hpool4 [1, 1]	hpool4 [1, 1]	hpool4 [1, 1]
lnorm4	lnorm4	lnorm4
conv5 [6, 6, 512]	conv5 [6, 6, 512]	conv5 [6, 6, 512]
relu5	relu5	relu5
hpool5 [1, 1]	hpool5 [1, 1]	hpool5 [1, 1]
lnorm5	lnorm5	lnorm5
conv6 [8, 8, 512]	conv6 [8, 8, 512]	conv6 [8, 8, 512]
relu6	relu6	relu6
hpool6 [2, 4]	hpool6 [2, 4]	hpool6 [2, 4]
lnorm6	lnorm6	lnorm6
flatten	flatten	flatten
fc0 [512]	fc0 [512]	fc0 [512]
relu_fc0	relu_fc0	relu_fc0
norm_fc0	norm_fc0	norm_fc0
dropout	dropout	dropout
fc [517, 433, 794]	fc [517, 433, 794]	fc [517, 433, 794]

Supplementary Table 2. Details of the three neural network architectures used for the sound localization task

**arch01**	**arch02**	**arch03**
conv0 [1, 8, 32]	conv0 [2, 8, 32]	conv0 [1, 4, 32]
mpool0 [1, 1]	mpool0 [1, 1]	mpool0 [1, 1]
relu0	relu0	relu0
lnorm0	lnorm0	lnorm0
conv1 [1, 64, 32]	conv1 [3, 16, 32]	conv1 [3, 32, 32]
mpool1 [1, 1]	mpool1 [1, 1]	mpool1 [1, 8]
relu1	relu1	relu1
lnorm1	lnorm1	lnorm1
conv2 [1, 64, 32]	conv2 [2, 4, 32]	conv2 [3, 32, 64]
mpool2 [1, 8]	mpool2 [1, 8]	mpool2 [1, 1]
relu2	relu2	relu2
lnorm2	lnorm2	lnorm2
conv3 [2, 4, 64]	conv3 [3, 16, 64]	conv3 [1, 8, 64]
mpool3 [2, 4]	mpool3 [1, 1]	mpool3 [1, 4]
relu3	relu3	relu3
lnorm3	lnorm3	lnorm3
conv4 [3, 8, 128]	conv4 [1, 8, 64]	conv4 [3, 8, 64]
mpool4 [1, 1]	mpool4 [1, 4]	mpool4 [1, 1]
relu4	relu4	relu4
lnorm4	lnorm4	lnorm4
conv5 [3, 32, 128]	conv5 [3, 8, 128]	conv5 [1, 2, 64]
mpool5 [1, 4]	mpool5 [1, 4]	mpool5 [1, 1]
relu5	relu5	relu5
lnorm5	lnorm5	lnorm5
conv6 [3, 4, 256]	conv6 [2, 2, 128]	conv6 [2, 2, 64]
mpool6 [1, 1]	mpool6 [1, 2]	mpool6 [2, 4]
relu6	relu6	relu6
lnorm6	lnorm6	lnorm6
conv7 [3, 8, 256]	conv7 [3, 2, 256]	conv7 [2, 4, 128]
mpool7 [1, 2]	mpool7 [1, 2]	mpool7 [1, 1]
relu7	relu7	relu7
lnorm7	lnorm7	lnorm7
flatten	conv8 [1, 8, 512]	conv8 [1, 8, 128]
fc0 [512]	mpool8 [1, 2]	mpool8 [1, 1]
relu_fc0	relu8	relu8
norm_fc0	lnorm8	lnorm8
dropout	flatten	conv9 [3, 2, 128]

fc [504]	fc0 [512]	mpool9 [1, 4]
	relu_fc0	relu9
	norm_fc0	lnorm9
	dropout	flatten
	fc [504]	fc0 [512]
		relu_fc0
		norm_fc0
		dropout
		fc [504]
