# Supplementary material for: A deep learning framework for understanding cochlear implants
Source: bioRxiv. 2025 Jul 21:2025.07.16.665227. Preprint. [Version 1] doi: 10.1101/2025.07.16.665227 (PMC12330565; doi:10.1101/2025.07.16.665227)
Supplement: Supplementary file 1 [file NIHPP2025.07.16.665227v1-supplement-1.pdf]

Supplementary Figure 1. Comparison of word recognition by normal hearing model and normal hearing humans, for speech in noise and noise-vocoded speech.

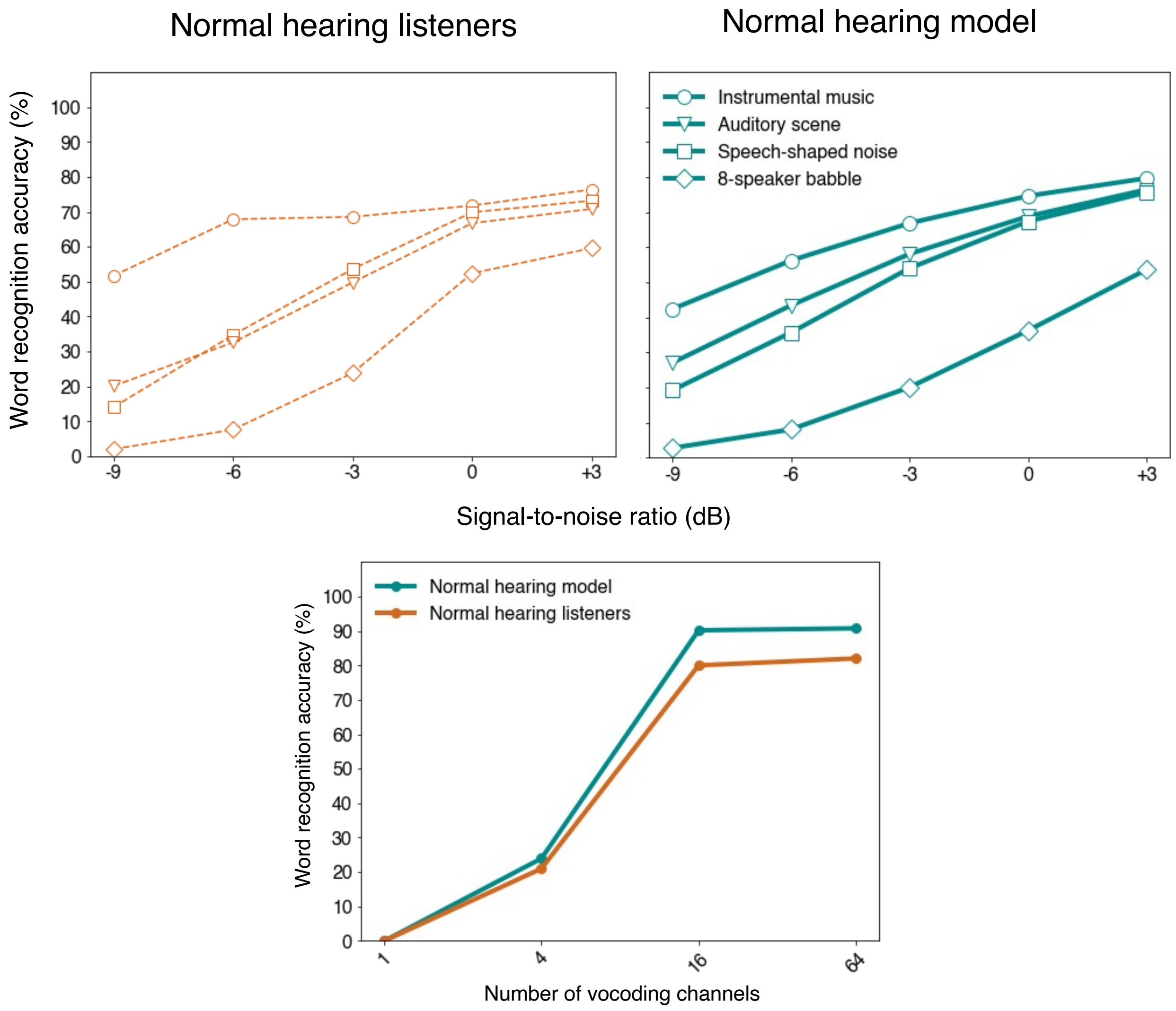

Supplementary Figure 2. ITD-ILD bias for CI models with different sound coding strategies

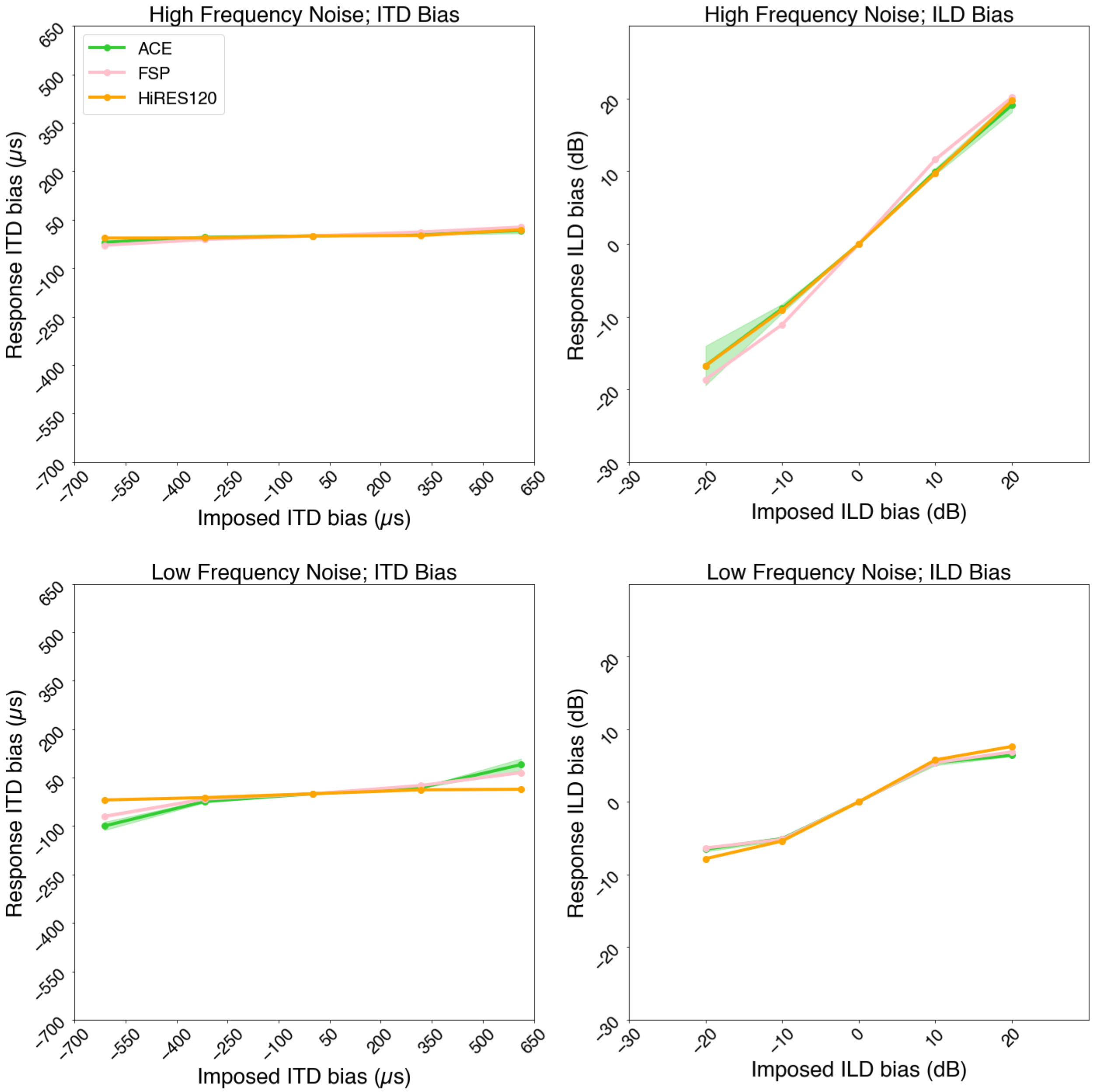

Supplementary Table 1. Details of the three neural network architectures used for the word recognition task

arch0_0000	arch0_0001	arch0_0002
input_lnorm	input_lnorm	input_lnorm
conv0 [2, 42, 32]	conv0 [1, 84, 32]	conv0 [4, 21, 32]
relu0	relu0	relu0
hpool0 [2, 4]	hpool0 [2, 4]	hpool0 [2, 4]
lnorm0	lnorm0	lnorm0
conv1 [2, 18, 64]	conv1 [2, 18, 64]	conv1 [2, 18, 64]
relu1	relu1	relu1
hpool1 [2, 4]	hpool1 [2, 4]	hpool1 [2, 4]
lnorm1	lnorm1	lnorm1
conv2 [6, 6, 128]	conv2 [6, 6, 128]	conv2 [6, 6, 128]
relu2	relu2	relu2
hpool2 [1, 4]	hpool2 [1, 4]	hpool2 [1, 4]
lnorm2	lnorm2	lnorm2
conv3 [6, 6, 256]	conv3 [6, 6, 256]	conv3 [6, 6, 256]
relu3	relu3	relu3
hpool3 [1, 4]	hpool3 [1, 4]	hpool3 [1, 4]
lnorm3	lnorm3	lnorm3
conv4 [8, 8, 512]	conv4 [8, 8, 512]	conv4 [8, 8, 512]
relu4	relu4	relu4
hpool4 [1, 1]	hpool4 [1, 1]	hpool4 [1, 1]
lnorm4	lnorm4	lnorm4
conv5 [6, 6, 512]	conv5 [6, 6, 512]	conv5 [6, 6, 512]
relu5	relu5	relu5
hpool5 [1, 1]	hpool5 [1, 1]	hpool5 [1, 1]
lnorm5	lnorm5	lnorm5
conv6 [8, 8, 512]	conv6 [8, 8, 512]	conv6 [8, 8, 512]
relu6	relu6	relu6
hpool6 [2, 4]	hpool6 [2, 4]	hpool6 [2, 4]
lnorm6	lnorm6	lnorm6
flatten	flatten	flatten
fc0 [512]	fc0 [512]	fc0 [512]
relu_fc0	relu_fc0	relu_fc0
norm_fc0	norm_fc0	norm_fc0
dropout	dropout	dropout
fc [517, 433, 794]	fc [517, 433, 794]	fc [517, 433, 794]

Supplementary Table 2. Details of the three neural network architectures used for the sound localization task

**arch01**	**arch02**	**arch03**
conv0 [1, 8, 32]	conv0 [2, 8, 32]	conv0 [1, 4, 32]
mpool0 [1, 1]	mpool0 [1, 1]	mpool0 [1, 1]
relu0	relu0	relu0
lnorm0	lnorm0	lnorm0
conv1 [1, 64, 32]	conv1 [3, 16, 32]	conv1 [3, 32, 32]
mpool1 [1, 1]	mpool1 [1, 1]	mpool1 [1, 8]
relu1	relu1	relu1
lnorm1	lnorm1	lnorm1
conv2 [1, 64, 32]	conv2 [2, 4, 32]	conv2 [3, 32, 64]
mpool2 [1, 8]	mpool2 [1, 8]	mpool2 [1, 1]
relu2	relu2	relu2
lnorm2	lnorm2	lnorm2
conv3 [2, 4, 64]	conv3 [3, 16, 64]	conv3 [1, 8, 64]
mpool3 [2, 4]	mpool3 [1, 1]	mpool3 [1, 4]
relu3	relu3	relu3
lnorm3	lnorm3	lnorm3
conv4 [3, 8, 128]	conv4 [1, 8, 64]	conv4 [3, 8, 64]
mpool4 [1, 1]	mpool4 [1, 4]	mpool4 [1, 1]
relu4	relu4	relu4
lnorm4	lnorm4	lnorm4
conv5 [3, 32, 128]	conv5 [3, 8, 128]	conv5 [1, 2, 64]
mpool5 [1, 4]	mpool5 [1, 4]	mpool5 [1, 1]
relu5	relu5	relu5
lnorm5	lnorm5	lnorm5
conv6 [3, 4, 256]	conv6 [2, 2, 128]	conv6 [2, 2, 64]
mpool6 [1, 1]	mpool6 [1, 2]	mpool6 [2, 4]
relu6	relu6	relu6
lnorm6	lnorm6	lnorm6
conv7 [3, 8, 256]	conv7 [3, 2, 256]	conv7 [2, 4, 128]
mpool7 [1, 2]	mpool7 [1, 2]	mpool7 [1, 1]
relu7	relu7	relu7
lnorm7	lnorm7	lnorm7
flatten	conv8 [1, 8, 512]	conv8 [1, 8, 128]
fc0 [512]	mpool8 [1, 2]	mpool8 [1, 1]
relu_fc0	relu8	relu8
norm_fc0	lnorm8	lnorm8
dropout	flatten	conv9 [3, 2, 128]

fc [504]	fc0 [512]	mpool9 [1, 4]
	relu_fc0	relu9
	norm_fc0	lnorm9
	dropout	flatten
	fc [504]	fc0 [512]
		relu_fc0
		norm_fc0
		dropout
		fc [504]
